# Podocyturia: an earlier biomarker of cardiovascular outcomes

**DOI:** 10.1038/s41598-022-26162-6

**Published:** 2022-12-13

**Authors:** Assaad A. Eid, Robert H. Habib, Omar Chehab, Nour Al Jalbout, Hani Tamim, Maha Makki, Martine El Bejjani, Joao Lima, Kamal F. Badr

**Affiliations:** 1 Department of Anatomy, Cell Biology and Physiology, American University of Beirut, Beirut, Lebanon; 2 Vascular Medicine Program, Faculty of Medicine, American University of Beirut, Riad El Solh, P.O. Box: 11-0236, Beirut, 1107 2020 Lebanon; 3 Department of Internal Medicine, American University of Beirut, Beirut, Lebanon; 4 Department of Cardiology, Johns Hopkins University School of Medicine, Baltimore, MD USA; 5 Department of Emergency Medicine, Massachusetts General Hospital, Boston, MA USA; 6 Clinical Research Institute, Faculty of Medicine, American University of Beirut, Beirut, Lebanon

**Keywords:** Biomarkers, Cardiology, Endocrinology

## Abstract

Urinary podocin and nephrin mRNAs (podocyturia), as candidate biomarkers of endothelial/podocyte injury, were measured by quantitative PCR in Type II diabetics with normal albumin excretion rates (AER) at baseline, at 3–4 years, and at 7 years. Development of cardiovascular disease (CVD) was collected as outcome. Visit 1 podocyturia was significantly higher in subjects who subsequently developed CVD versus those who did not. Visit 1 AER terciles exhibited similar time to CVD, in contrast with stepwise and substantial increases in CVD events predicted by Visit 1 podocyturia terciles. Covariate-adjusted hazard ratios were highest for podocin, intermediate for nephrin mRNAs, and lowest for AER. Podocyturia was also measured in patients with and without significant coronary obstruction, and in 480 normoalbuminuric subjects at the enrolment visit to the Multi-Ethnic Study of Atherosclerosis (MESA). Podocyturia > 3 × 10^6^ copies was associated with presence of obstructive coronary artery disease. In the MESA population, Visit 1 podocyturia was significantly higher in men, subjects with elevated BMI, and those with Type II DM. Conclusions: Podocyturia may be an earlier predictor of cardiovascular events than moderate albuminuria; it is significantly higher in patients with obstructive coronary artery disease, and in subjects with established risk factors for CVD.

## Introduction

Increased albumin excretion rates (AER) are known to independently predict worsening cardiovascular disease (CVD) and outcomes, including increased mortality, in a wide range of clinical settings^1–6^. A large body of high-quality evidence has established this direct relation of systematically worse clinical outcomes with increasing levels of AER to be derivative of vascular endothelial injury (disease) that is observed in both diabetic and non-diabetic individuals^1–6^. Ruggegnenti and colleagues demonstrated, in a large population of initially “normoalbuminuric” diabetics, that increasing AER is associated with a continuous nonlinear relationship with CVD development over a mean follow-up period of 9.2 years. Even minimal increases in measurable albuminuria (< 2 µg/min) conferred a significant increase in risk^6^. Because it is such a powerful and independent predictor of CVD, “moderate albuminuria” (previously “microalbuminuria”) has been incorporated as a significant risk factor in several national and international guidelines for the treatment of hypertension and other vascular diseases (7). It is also used in the estimation of risk of mortality in Type II diabetic patients^7^.

Numerous reviews focused on the link between urinary albumin excretion and cardiovascular risk implicate endothelial cell vascular injury as the common culprit mechanism^8–10^. This injury simultaneously affects both the systemic and renal vasculature, but is detectable only through its measurable functional consequence at the glomerulus as increased albumin filtration, ultimately leading to progressive albuminuria. However, the active re-uptake of filtered albumin by the proximal tubule may largely mask the presence of this injury^8,9^. Albuminuria may thus be a ‘late’ reflection of endothelial injury that is detectable only in relatively advanced vascular disease.

Increased albumin filtration is invariably accompanied by injury to subjacent glomerular epithelial cells (podocytes); multiple studies have strongly correlated the shedding of podocytes (podocyturia) with albuminuria^11–14^. We hypothesized that, unlike albumin excretion, which is masked by proximal tubule reabsorption, podocyturia is detectable in the urine at very early stages of vascular endothelial injury. Accordingly, an increase in urinary podocytes may provide an early biomarker of systemic endothelial dysfunction well before the emergence of moderate albuminuria, and would hence facilitate earlier diagnosis of affected individuals with expected improvement in CVD outcomes. In this study, we sought to test and validate this hypothesis in multiple distinct clinical cohorts.

## Results

### Development cohort A

**Figure 1:**
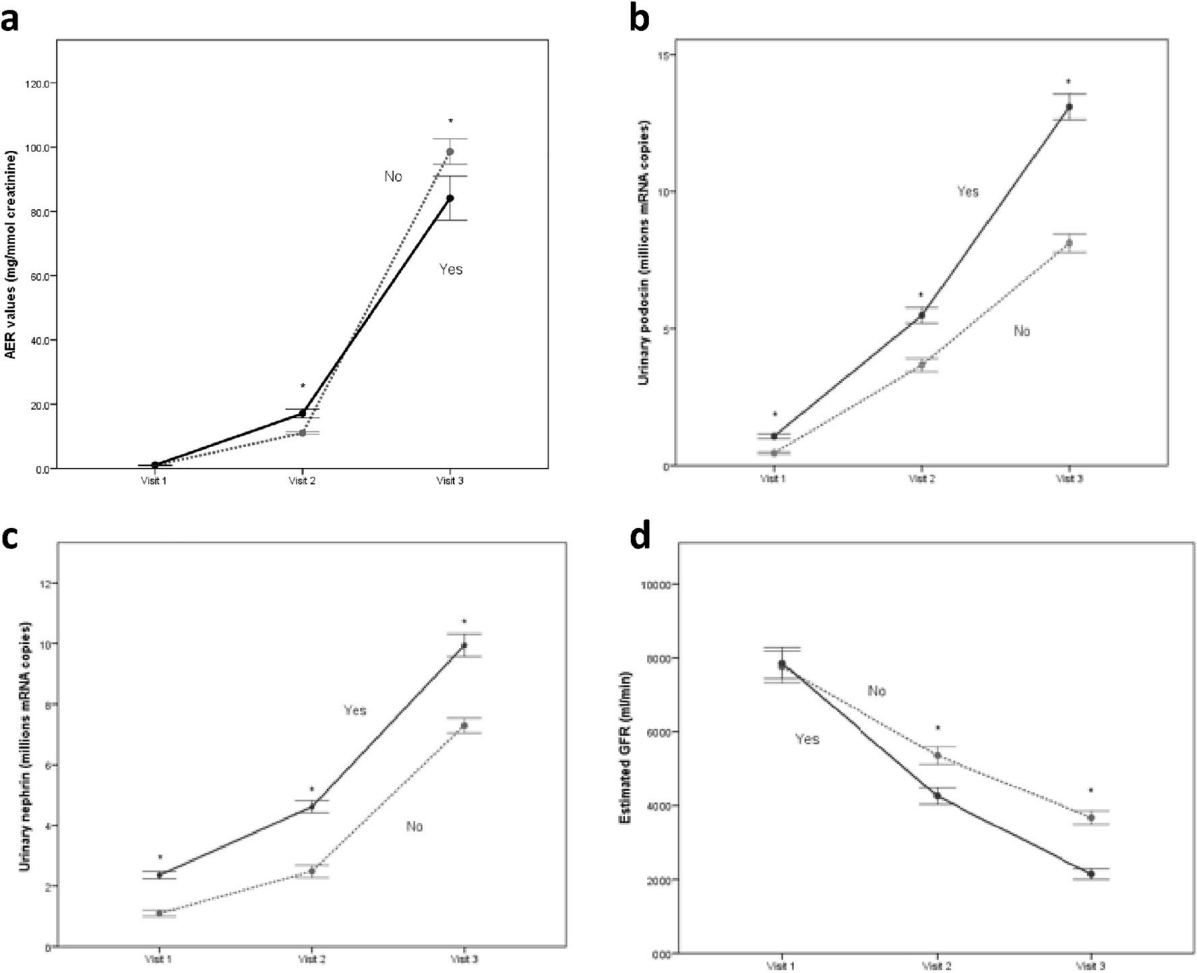
Alteration in urinary biomarkers sampled at different visits. Values for AER (**a**) urinary podocin (**b**) urinary nephrin (**c**) RNA copies and estimated GFR (CKD-epi equation; **d**) across Visits 1–3 in Type II diabetic subjects who developed (Yes; solid line) and did not develop (No; dotted line) CVD. *: statistically different (see text for details).

Table 1 summarizes subject characteristics, clinical and laboratory data, and cardiovascular outcomes by baseline podocyturia marker sub-groups. Briefly, at Visit 1, all 106 subjects in this diabetic study population were free of any known CVD and all had “normal” albuminuria levels [AER < 2.26 mg/mmol; median (IQR) = 1.07 (0.74–1.19)]. Figure 1 summarizes the change of all three urinary biomarkers (albumin, podocin mRNA, nephrin mRNA) sampled at three visits (1, 2 and 3) that spanned a median follow-up period of 7.1 years. All three urine biomarkers increased over time in all patients in parallel with a gradual decline in eGFR, consistent with progression of diabetic vascular injury. Figure 1. Importantly, urinary podocin and nephrin levels were already significantly higher at Visit 1 in subjects who subsequently developed CVD versus those who did not, whereas AERs were indistinguishable. Figure 1. At Visit 2 (at 3 to 4 years), AER had increased substantially, and this was followed by an additional marked increase at Visit 3 in both groups. The corresponding urinary podocin mRNA, and nephrin mRNA levels also increased progressively between Visits 1 and 3 (all *p* < 0.001). Figure 1. Comparison of the rate of increase in biomarkers from Visit 1 to Visit 2, and from Visit 2 to Visit 3 revealed a clearly greater rate of rise for all three markers in CVD versus No CVD patients. Figure 1.

**Table 1:** Comparisons of subject characteristics, clinical and laboratory data for baseline podocyturia marker sub-groups.

Factor	Baseline podocin terciles (visit 1)						Baseline nephrin terciles (visit 1)					
	Low (n = 36)		Intermediate (n = 35)		High (n = 35)		Low (n = 36)		Intermediate (n = 35)		High (n = 35)	
Continuous	Mean	SD	Mean	SD	Mean	SD	Mean	SD	Mean	SD	Mean	SD
Age (years)	47	8.2	45.6	10.0	46.9	8.6	46.4	8.1	46	9.7	47.1	9.0
BMI (kg/m^2^)	27.8	2.90	27.9	3.4	28.2	2.7	28.2	2.7	27.9	3.6	27. 8	3.9
BSA (m^2^)	1.98	0.13	1.97	0.22	1.87	0.26	1.99	0.13	1.94	0.23	1.9	0.26
SBP (mmHg)	131	9	126	9	133	9^b^	130	9	123	9^ a^	136	7^ a,b^
DBP (mmHg)	70	8	66	7	71	6^b^	69	7	66	8	72	6^ b^
TG (mmol/l)	2.0	0.7	1.5	0.6^a^	1.4	0.4^a^	1.9	0.7	1.6	0.7	1.4	0.5^ a,b^
Total Cholesterol (mmol/l)	6.9	1.3	8.1	8.0	6.7	1.4	7.3	1.2	7.6	0.8	6.9	1.4
HDL (mmol/l)	1.09	0.37	1.11	0.39	0.97	0.30	1.13	0.36	1	0.41	1.04	0.28
LDL (mmol/l)	3.7	0.7	3.4	0.6	3.2	0.7^a^	3.7	0.7	3.3	0.7	3.4	0.6
HbA1C (%)	7.4	0.5	7.2	0.5	7.4	0.5	7.3	0.5	7.2	0.5	7.5	0.4^ b^
Serum creatinine (mmol/L)	97	17	97	9	106	9^b^	97	17	97	18	106	9^ a,b^
Podocin mRNA (10^6^ copies)	0.39	0.07	0.68	0.13^a^	1.27	0.17^a,b^	0.45	0.11	0.68	± 0.28^ a^	1.2	0.27^ a,b^
Nephrin mRNA (10^6^ copies)	1.1	0.35	1.7	0.71^a^	2.5	0.37^a,b^	0.9	0.2	1.7	± 0.3^ a^	2.6	0.2^ a,b^
AER (mg/mmol)	0.95	0.26	0.90	0.24	1.15	0.20^b^	0.94	0.25	0.92	0.25	1.13	0.23^ a,b^

Categorical	N	(%)	N	(%)	N	(%)	N	(%)	N	(%)	N	(%)
Male	12	33%	16	46%	18	51%	14	39%	14	40%	18	51%
Smoking	31	86%	32	91%	29	83%	32	89%	29	83%	31	89%
Hypertension	5	14%	8	23%	4	11%	8	22%	3	8.6%	6	17%
Oral hypoglycemics	22	61%	15	43%	20	57%	20	56%	13	37%	24	69%^ b^
Insulin	31	86%	29	83%	30	86%	33	92%	27	77%	30	86%
ACE inhibitor	11	31%	8	23%	16	46%	12	33%	5	14%	18	51%^ b^
angiotensin receptor blocker	10	28%	4	11%	7	20%	7	19%	4	11%	10	29%
Statin	9	25%	9	26%	16	46%	9	25%	9	26%	16	46%
**Outcomes at 7 years**												
Cerebrovascular disease	1	3%	19	54%^ a^	35	100% ^a,b^	2	5.6%	21	60%	34	97%^ b^
Coronary artery disease	1	3%	12	34% ^a^	26	74%^ a,b^	2	5.6%	14	40%	25	71%^ b^
Atrial fibrillation	1	3%	14	40% ^a^	23	66%^ a^	2	5.6%	14	40%	24	69%^ b^
Peripheral vascular disease	1	3%	9	26%^ a^	8	23% ^a^	2	5.6%	7	20%	11	31%

^a^*p* < 0.05 compared to Low tercile; ^b^*p* < 0.05 compared to Intermediate tercile.

#### Time to cardiovascular event analysis

**Figure 2:**
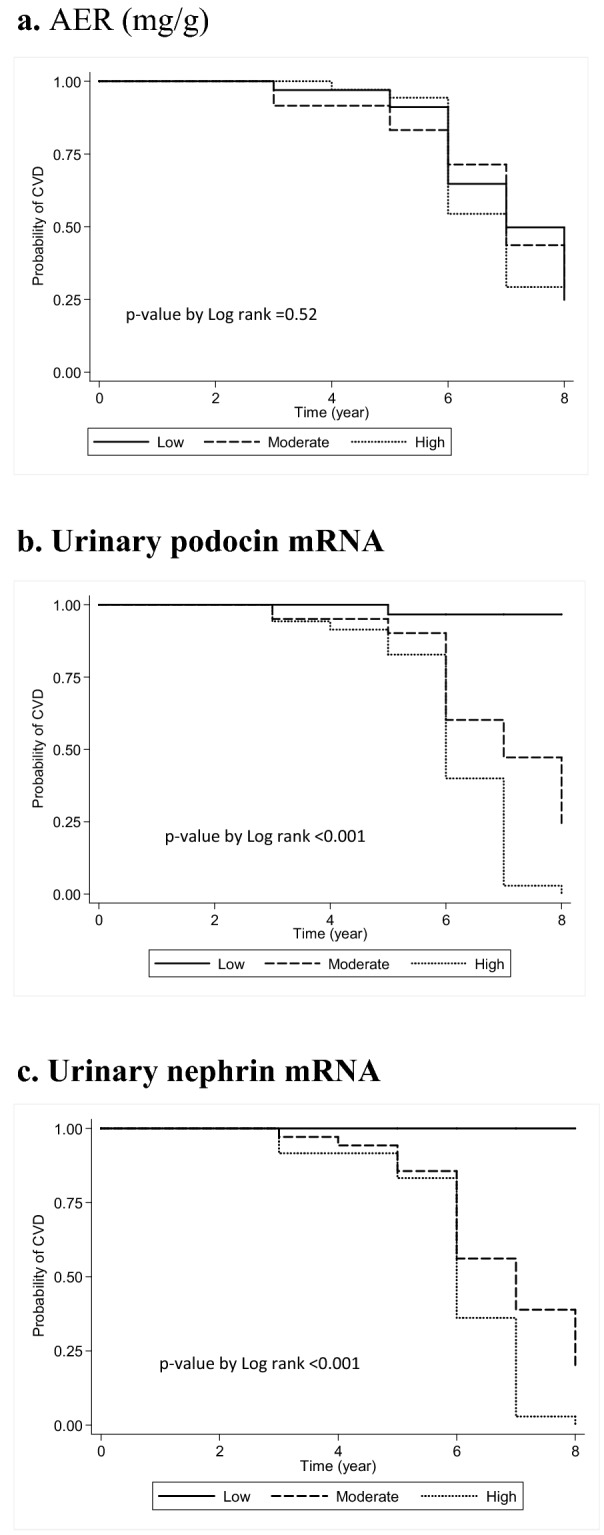
Time-to-Event analysis comparing the derived subgroups for the different biomarkers. Probability of CVD analyzed using Kaplan–Meier survival analysis with comparisons among the levels of AER (a; *p* value by Log rank = 0.52), urinary podocin mRNA (b; *p* value by Log rank < 0.0001) and urinary nephrin mRNA (c; *p* value by Log rank < 0.0001) using log-rank test.

To ascertain the early discriminatory power of each of the urinary biomarkers in predicting CVD, subjects were categorized into 3 subgroups based on their Visit 1 levels using specific cutoff values for AER (< 0.79 (Low); 0.79–1.13 (med) and > 1.13 (high) mg/mmol), or using tercile groups in the case of podocin and nephrin mRNA. Comparisons of subject characteristics, clinical data and laboratory values for the podocin and nephrin sub-groups are detailed in Table 1. Development of cardiovascular disease after enrollment was then investigated as a Time-to-Event analysis comparing the derived subgroups for each of the three biomarkers. Figure 2. This analysis indicated that, low, medium and high levels of AER at baseline, albeit all within normal levels (< 2.26 mg/mmol), do not separate patients who later developed versus those who did not develop CVD within the 7-year follow-up period Fig. 2; Table 2; *p* = 0.127. This was in sharp contrast with the results obtained with either podocin or nephrin mRNA levels. Figure 2, Table 2. Increasing levels of podocin and nephrin levels at baseline were highly significantly predictive of development of CVD (both *p* < 0.001), as well as the time to CVD diagnosis or event occurrence. To complement this analysis, we also analyzed biomarker levels as continuous variables: the associated covariate-adjusted hazard ratios by multivariate Cox Regression were highest in case of podocin mRNA [HR = 15.9 (6.1–41.8); *p* < 0.001], intermediate for nephrin mRNA [HR = 7.61 (3.75–15.5); *p* < 0.001] and lowest for AER [HR = 1.17 (1.01–1.36); *p* = 0.041]. Table 2.

**Table 2:** Hazard Ratios for the development of cardiovascular disease derived for all three urinary biomarkers considered as continuous or categorical biomarkers.

Biomarker	N	B	SE	*p* value	Adjusted HR (95% CI)
**Continuous values**					
AER (mcg/mg)	106	0.155	0.076	0.041	1.17 (1.01–1.36)
Podocin mRNA (10^6^ copies)	106	2.767	.492	< 0.001	15.9 (6.1–41.8)
Nephrin mRNA (10^6^ copies)	106	2.030	.361	< 0.001	7.61 (3.75–15.5)
**Two categories**^** #**^					
AER	52/54	0.502	0.329	0.127	1.65 (0.87–3.14)
Podocin mRNA	53/53	2.507	0.506	< 0.001	12.3 (4.55–33.1)
Nephrin mRNA	52/54	2.767	0.520	< 0.001	15.9 (5.74–44.0)
**Three categories**					
AER				0.289	
Low	34				1.0 (ref)
Intermediate	32	0.426	0.430	0.322	1.53 (0.66–3.55)
High	40	0.610	0.390	0.118	1.84 (0.86–3.95)
Podocin terciles				< 0.001	
Low	36			< 0.001	1.0 (ref)
Intermediate	35	3.003	1.049	0.004	20.1 (2.58–157.4)
High	35	4.086	1.039	< 0.001	59.5 (7.8–456.2)
Nephrin terciles *		*	*	*	*

N = number of subjects; B (SE) = cox regression coefficient (standard error); HR (95% CI) = hazard ratio (95% confidence interval). Data adjusted for the following Visit 1 factors: gender, age, body mass index, smoking, hypertension (yes/no), statin use (yes/no), systolic and diastolic blood pressure, triglyceride, low and high density lipoprotein, and hemoglobin A1c levels.

^#^Biomarker groups divided to sub-cohorts based on the median visit 1 value.

*Not calculated since no subjects in the low nephrin tercile group had documented CVD during follow-up.

The percent of patients being treated with ACE-I/ARB in the group that developed CVD was 38% on Visit 1 and had increased to 61% by visit 3. These values were less than those in the group that did not develop CV events over the same period of time (44% and 66%, respectively). While these findings are consistent with those demonstrated in Cohort B (see below) and in Reference 6, the differences did not reach statistical significance at either time point.

### Validation Cohort B

The demographic and clinical characteristics of patients and their association with obstructive CAD and podocyturia mRNA (podocin) elevation are listed in Table 3. Patients that were found to have significant CAD were more likely to be older (mean, 65.30 [± 10.27] vs 61.17 [± 8.64], *p* = 0.03) and of male (85.5% vs 14.5%, *p* = 0.01). In addition, those with obstructive CAD had a higher prevalence of hypertension (77%, *p* = 0.04), lower levels of LDL (*p* = 0.01), HDL (*p* = 0.04), and total cholesterol (*p* = 0.001), previous history of myocardial infarction (MI), percutaneous coronary intervention (PCI) or coronary artery bypass graft (CABG) (48%, *p* = 0.005). Also, those with evidence of CAD were more likely to be on insulin (*p* = 0.02) and oral antidiabetic (*p* = 0.04) medications.

**Table 3:** Demographical and characteristic summary of the study sample based on obstructive coronary artery disease and podocin mRNA.

		Vessel			Podocin mRNA (10^6^ copies/mg creatinine)		
	ALL N = 108	Non-Obstructive N = 46	Obstructive N = 62	*p* value	< 3 N = 49	≥ 3 N = 59	*p* value
**Demographics**							
Gender (%)							
Male	83 (77)	30 (65)	53 (85.5)	**0.01**	36 (73.5)	47 (80)	0.45
Female	25 (23)	16 (35)	9 (14.5)		13 (26.5)	12 (20)	
Age, mean (± SD) (years)	63.54 ± 9.71	61.17 ± 8.64	65.30 ± 10.27	**0.03**	62.87 ± 9.40	64.10 ± 10.15	0.52
< 63 (%)	54 (50)	28 (60.9)	26 (42)	**0.05**			
≥ 63 (%)	54 (50)	18 (39.1)	36 (58)				
**BMI**, mean (± SD) (kg/m^2^)	29.89 ± 4.81	30.60 ± 5.25	29.34 ± 4.42	0.22	30.36 ± 4.84	29.53 ± 4.80	0.42
**Laboratory, mean (± SD)**							
Creatinine (mmol/L)	0.93 ± 0.34	0.91 ± 0.46	0.95 ± 0.21	0.54	0.85 ± 0.19	0.99 ± 0.42	**0.05**
eGFR (mL/min/1.73 m^2^)	83.1 ± 18.57	85.68 ± 18.76	81.26 ± 18.40	0.29	86.24 ± 16.13	80.85 ± 20.01	0.20
Triglyceride (mmol/l)	157.5 ± 81.72	170.83 ± 98.41	149.05 ± 69.29	0.62	164.67 ± 68.39	151.94 ± 91.29	0.55
LDL-C (mmol/l)	105.54 ± 44.02	124.38 ± 47.72	94.51 ± 38.13	**0.01**	106.14 ± 49.45	105.06 ± 39.84	0.92
HDL-C (mmol/l)	41.95 ± 13.86	47.46 ± 15.83	38.56 ± 11.45	**0.04**	43.07 ± 14.89	41.11 ± 13.20	0.58
Total Cholesterol (mmol/l)	164.35 ± 50.16	192.22 ± 56.95	148.33 ± 38.07	**0.001**	170.14 ± 57.79	159.71 ± 43.44	0.42
Ratio LDL/HDL	4.40 ± 1.97	4.55 ± 2.15	4.32 ± 1.88	0.73	4.45 ± 2.03	4.38 ± 1.96	0.88
Glucose (mg/dl)	145.27 ± 68.20	135.38 ± 61.45	153.92 ± 73.81	0.16	144.48 ± 69.62	145.96 ± 68.43	0.94
HBA1C (%)	6.72 ± 1.55	6.33 ± 1.21	6.89 ± 1.66	0.42	6.2 ± 1.18	7.11 ± 1.69	**0.015**
BUN (mg/dL)	18.44 ± 7.59	18.17 ± 5.12	18.64 ± 9.07	0.80	16.92 ± 6.03	19.57 ± 8.48	0.15
Podocin mRNA, mean (± SD) (10^6^ copies)	4.88 ± 5.30	4.56 ± 6.24	5.12 ± 4.52	0.59	–	–	–
≤ 3 (%)	50 (46)	26 (56.5)	23 (37)	**0.04**			
> 3 (%)	58 (54)	20 (43.5)	39 (63)			–	
Obstructive Vessels, mean (± SD)	–	–	–		1.12 ± 1.47	1.89 ± 2.00	**0.03**
Normal (%)	–	–	–		26 (53)	20 (34)	**0.04**
Abnormal (%)	–	–	–		23 (47)	39 (66)	
0 Vessel					26 (53)	20(34)	0.26
1 Vessel					8 (16)	14 (24)	
2 Vessels					9 (18)	15 (25)	
3 Vessels					6 (12)	10 (17)	
Hemoglobin (g/dl)	13.48 ± 1.72	13.47 ± 1.67	13.50 ± 1.77	0.93	13.52 ± 1.45	13.45 ± 1.92	0.85
Hematocrit (%)	39.86 ± 6.19	39.47 ± 7.45	40.17 ± 5.01	0.60	40.63 ± 3.84	39.23 ± 7.57	0.30
**Medications, yes (%)**							
Insulin	11 (10)	1 (2)	10 (16)	**0.02**	2 (4)	9 (15)	0.11
Oral antidiabetics	36 (33)	10 (22)	26 (42)	**0.04**	12 (24.5)	24 (41)	0.08
Lipid lowering statin	63 (58)	30 (65)	33 (53)	0.21	28 (57)	35 (59)	0.82
Lipid lowering non statin	11 (10)	5 (11)	6 (10)	1.00	4 (8)	7 (12)	0.75
P2Y12 inhibitors	33 (31)	11 (24)	22 (35.5)	0.20	13(26.5)	20 (34)	0.41
Vitamin K antagonist	3 (3)	2 (4)	1 (2)	0.57	2 (4)	1 (1.7)	0.59
Ace inhibitor /ARB	49 (45)	17 (37)	32 (52)	0.13	23 (47)	26 (44)	0.76
Aspirin	71 (66)	26 (56.5)	45 (73)	0.08	30 (61)	41 (69.5)	0.37
Diuretics	19 (18)	11 (24)	8 (13)	0.14	9 (18)	10 (17)	0.85
**Risk, yes ()**							
Cardiac Arrest, 24 h before presentation	2 (2)	1 (2)	1 (2)	1.00	1 (2)	1 (2)	1.00
Diabetes status	44 (41)	10 (22)	34 (55)	**0.001**	14 (29)	30 (51)	**0.02**
Family history CAD	13 (12)	6 (13)	7 (11)	0.77	4 (8)	9 (15)	0.26
**Renal status**							
Normal	95 (88)	43 (93.5)	52 (84)	0.08	45 (92)	50 (85)	0.43
Insufficiency	12 (11)	2 (4)	10 (16)		4 (8)	8 (14)	
Failure—no dialysis	1 (1)	1 (2)	0 (0)		0 (0)	1 (2)	
Smoker	40 (37)	16 (35)	24 (39)	0.68	19 (39)	21(36)	0.73
Hypertension	75 (69)	27 (59)	48 (77)	**0.04**	33 (67)	42 (71)	0.67
Dyslipidemia	61 (56.5)	29 (63)	32 (52)	0.24	30 (61)	31 (52.5)	0.36
Cerebrovascular Disease	3 (3)	2 (4)	1 (2)	0.57	0 (0)	3 (5)	0.25
Peripheral Arterial Disease	3 (3)	0 (0)	3 (5)	0.26	1 (2)	2 (3)	1.00
Chronic Lung Disease	9 (8)	3 (6.5)	6 (10)	0.73	2 (4)	7 (12)	0.18
Prior Heart Failure	11 (10)	5 (11)	6 (10)	1.00	5 (10)	6 (10)	1.00
MI/PCI/CABG	40 (37)	10 (22)	30 (48)	**0.005**	17 (35)	23 (39)	0.65
Prior Cath	44 (41)	14 (30)	30 (48)	0.06	19 (39)	25 (42)	0.70
Prior valve surgery procedure	5 (5)	4 (9)	1 (2)	0.16	3 (6)	2 (3)	0.66

Significant values are in bold.

Importantly, patients with a podocyte-specific protein mRNA level of > 3 (× 10^6^) were more likely to be associated with having obstructive coronary artery disease (63% vs 43.5%, *p* value = 0.04) as well as more likely to have a diagnosis of diabetes (51% vs 29%, *p* = 0.02), a greater number of diseased coronary vessels (1.89 ± 2.00 vs 1.12 ± 1.47, *p* = 0.03), a higher mean hemoglobin A1C level (7.11 ± 1.69 vs 6.2 ± 1.18, *p* = 0.015) and higher creatinine levels (0.99 ± 0.42 vs 0.85 ± 0.19, *p* = 0.05).

Significant predictors for CAD by multivariable logistic regression were gender (OR = 3.59; 95% CI 1.21–10.71, *p* = 0.02), diabetes (OR = 2.88; 95% CI 1.14–7.33, *p* = 0.03), and history of MI, PCI, or CABG (OR = 2.31; 95% CI, 0.90–5.96, *p* = 0.08) as illustrated in Table 1-Supplement.

A separate multivariate logistic analysis was performed in patients with elevated mRNA (> 3) and abnormal vessels and is presented in Table 2 Supplement. We chose 3 as the cutoff value, because podocin mRNA levels did not follow a normal distribution and were skewed to the left; therefore, the median was used as a measure of central tendency. When this association was stratified according to patients taking angiotensin-converting enzyme inhibitor (ACEi)/angiotensin receptor blocker (ARB), it was found that among those who were not taking ACEi/ARB, those with mRNA > 3 were found to have 3.31 more significant risk of abnormal vessel, as compared to those who are on ACEi/ARB (OR: 3.31, CI [1.13–9.69], *p* = 0.03 vs OR: 1.45, CI [0.44–4.71], *p* = 0.54; respectively). When the association was stratified according to those who had a history of MI, PCI, or CABG, it was found that among those patients, those with RNA > 3 were found to have 4.67 more significant risk of abnormal vessel, as compared to those with no history of CVD (OR: 4.67, CI [0.99–22.01], *p* = 0.05 vs OR: 1.63, CI [0.62–4.28], *p* = 0.32; respectively).

### Validation cohort C

Table 4 summarizes the demographic characteristics of the MESA sample included in this study stratified by podocin count terciles. There was an increased prevalence of diabetes (3.8% vs. 6.8% vs. 10.1%, *p* = 0.08) and higher BMI (27.09 ± 5.18 vs. 28.64 ± 4.84 vs. 29.46 ± 6.26, *p* = 0.0005) with increasing podocin tercile). Moreover, patients with type II diabetes mellitus had a significantly higher rate of absolute podocin (3.7737 ± 3.6283 vs. 2.2527 ± 1.6998, *p* < 0.0001) and nephrin counts (4.7039 ± 4.1761 vs. 2.5924 ± 1.7954, *p* < 0.0001) as well as a higher rate of podocin (4.5012 ± 5.2214 vs. 2.4211 ± 1.8751, *p* < 0.0001) and nephrin to creatinine ratio (5.6045 ± 5.2041 vs. 2.7894 ± 2.0667), *p* < 0.0001 compared to non-diabetics. Table 5.

**Table 4:** Characteristics of the MESA study population stratified by Podocin Tertiles.

	Lowest tertile	Intermediate tertile	Highest tertile	*p* value
Age, mean (± SD)	59.8** ± **9.66	59.78** ± **9.72	59.70** ± **9.83	0.511
Gender, Females	105 (65.6%)	83 (51.6%)	82 (51.6%)	0.014
Systolic blood pressure, mmHg (± SD)	124.02** ± **22.52	124.81** ± **18.06	120.09** ± **18.94	0.08
Body mass index, kg/m^2^ (± SD)	27.09** ± **5.18	28.64** ± **4.84	29.46** ± **6.26	0.0005
Education, low	48 (30%)	45 (28%)	46 (29%)	0.92
**Income**				
Low	37 (23.6%)	41 (26%)	40 (26%)	0.61
Middle	65 (41.4%)	68 (43%)	73 (47%)	
High	55 (35%)	49 (31%)	41 (27%)	
**Race**				
White, Caucasian	117 (73%)	97 (60.3%)	94 (59%)	0.12
Chinese American	7 (4.4%)	9 (5.6%)	14 (9%)	
Black, African American	15 (9.4%)	24 (15%)	20 (12.6%)	
Hispanic	21 (13.13%)	31 (19.3%)	31 (19.5%)	
Type 2 DM	6 (3.8%)	11 (6.8%)	33 (10.1%)	0.08
Anti-hypertensive mediation	39 (24.4%)	50 (31.1%)	44 (27.7%)	0.41
**Cigarette smoking**				
Never-smokers	82 (51.3%)	78 (48.5%)	74 (46.5%)	0.7
Former smokers	58 (36.3%)	63 (39.1%)	64 (40.3%)	
Current smokers	20 (12.5%)	20 (12.4%)	21 (13.2%)	
Creatinine, (mmol/L) (± SD)	0.93** ± **0.17	0.97** ± **0.19	0.95** ± **0.19	0.13
C-reactive protein, mg/L (± SD)	3.6** ± **4.7	4.2** ± **4.8	4.5** ± **7.6	0.38
Coronary artery calcium scores, Agatson units (± SD)	97.50** ± **315.14	115.02** ± **334.60	103.75** ± **360.93	0.9

**Table 5:** Mean copies of podocyte specific proteins: podocin and nephrin (absolute counts and when factored by mg creatinine in the urine) in the MESA sample stratified by diabetes status.

	Not diabetic N = 446		Diabetic N = 33		*p* value
	Mean	SD	Mean	SD	
Absolute Podocin count	2.2527	1.6998	3.7737	3.6283	< 0.0001
Podocin to creatinine ratio	2.4211	1.8751	4.5012	5.2214	< 0.0001
Absolute nephrin	2.5924	1.7954	4.7039	4.1761	< 0.0001
Nephrin to creatinine ratio	2.7894	2.0667	5.6045	5.2041	< 0.0001

## Discussion

Under physiologic (“normal”) conditions, an appreciable amount of albumin is filtered across glomerular epithelial cells (podocytes)^15–20^. In healthy children and adults, the amount of albumin appearing in the urine is the algebraic difference between filtered albumin and the amount retrieved by the S1 segment of the proximal tubule^15,16,19,21^. An increasingly large body of evidence in humans and other mammals assigns a greater role for proximal tubule albumin retrieval in determining urinary albumin excretion than previously appreciated^15,16,19,21^. Studies have established that proximal tubule albumin retrieval rates are highly regulated and are also influenced by total proximal tubule mass and disease states, including diabetes^16,19,21^. It therefore seems reasonable to assume that individuals vary widely in the proximal handling of filtered loads of albumin and fractional albumin retrieval. The tight coupling between AER and CV outcomes, however, suggests strongly that it is progressive glomerular and systemic microvascular injury, leading to an increase in filtered albumin, that underlies progressive albuminuria in these individuals. For urinary albumin to reflect filtered albumin, however, proximal tubule retrieval of albumin must be saturated. Only then would vascular injury (filtered albumin) correlate positively and tightly with urinary albumin, a relationship repeatedly demonstrated in numerous large population studies^1,2,4–6,21,22^. In an individual patient, however, the time elapsing between the onset of vascular injury and the saturation of proximal retrieval cannot be known; increased filtration of albumin may be present for years before urinary albumin levels begin to rise.

We examined the potential clinical usefulness of measuring podocyte shedding by quantitative PCR as an early biomarker of CVD in three separate cohorts, each designed to test the validity of this approach. Our development cohort (Cohort A) addressed the role of podocyturia as a predictive biomarker of future cardiovascular events. The purpose of Cohort B was to demonstrate the correlation of podocyturia with the severity of already established vascular disease, enforcing its proposed role as a faithful biomarker of vascular injury, not only during its pre-clinical phase, but also during its established later stages. The second validation cohort (C) tested, in a U.S. population of mixed ethnicities, whether podocyturia is indeed a biomarker that tracks with traditional risk factors for CVD.

Our developmental study addressed this hypothesis by targeting an earlier event in the vascular injury pathway, podocyte shedding, and assessing the capacity of podocyturia to predict CV outcomes before AER increases above the “normal” range. Podocyturia correlates strongly with proteinuria and renal functional deterioration in diabetic and non-diabetic individuals, and in patients with inflammatory and non-inflammatory glomerular diseases^23–27^, but it has not been previously evaluated as an early predictor of systemic vascular injury. Podocyturia is best measured by real-time PCR quantitation of mRNA for unique podocyte-specific proteins (such as nephrin, podocalyxin, podocin, synaptopodin, and others)^28,29^ .

Our first study population consisted of relatively young diabetics (mean age 47 years at Visit 1) who, despite being “normoalbuminuric”, carried other risk factors for progression of CVD, including a high proportion of smokers and overweight/obese individuals, coupled to relatively poor glycemic control. Not surprisingly, therefore, there were progressive increases in all three urinary biomarkers (AER and nephrin/podocin mRNA) between visits 1, 2 and 3, reflecting progressive vascular injury. Despite normal baseline AER levels, more than half of the subjects (56/106) developed cardiovascular disease within the 7-year follow-up period. Importantly, however, those that did versus did not develop CVD had similar baseline AER at enrollment, but significantly different podocin and nephrin mRNA. Figure 1. The rate of increase in biomarkers was accelerated in CVD versus No-CVD patients, supporting the notion that podocyte injury and albuminuria are indeed a reflection of progressive endothelial/vascular injury occurring silently and simultaneously in the cerebral, coronary, and systemic circulation.

Our data provide evidence for the potential superiority of nephrin/podocin mRNA over AER as a reliable marker of CV injury over time. Nephrin and podocin mRNA levels correlated well with each other, even as injury progressed over seven years, suggesting that podocyte shedding is indeed well-reflected by both measurements. More importantly, at all three visits, CVD patients had distinctly higher values of urinary podocin/nephrin mRNA than their No-CVD counterparts. That AER at Visit 1 did not correlate with nephrin or podocin mRNA, nor did it distinguish between CVD and No-CVD patients, strongly supports the notion that the major determinant of AER at this early stage of injury is not filtered albumin, but the fraction of it retrieved in the proximal tubule, as suggested by others^16,19,21,30^. At Visit 2, AER had increased to levels within the moderate albuminuria range (previously termed “microalbuminuria”) and indeed at this stage of injury, at which time filtered albumin contributes increasingly to the final value of AER, the well–established capacity of moderate albuminuria to distinguish CVD from No-CVD is evident and AER correlates well with both nephrin and podocin mRNA levels. By Visit 3, however, AER had increased to the macroalbuminuric range and is no longer relevant to identifying CV risk. Even at Visit 3, however, urinary podocin/nephrin mRNAs continue to distinguish the two patient groups.

The HR for development of CVD, particularly for podocin, was quite high (> 15). We can only ascribe this to the true predictive power of our biomarker. We believe the strength of this data reflects the combined power of four well-established facts: moderate albuminuria (previously microalbuminuria) is an exceptionally powerful predictor of CVD; podocyte shedding is a *sine qua non* for increased albumin filtration; the (now well-established) substantial retrieval of albumin along the proximal tubule delays the appearance of increased filtered albumin in the urine; even the smallest measurable increases in urinary albumin in “normoalbuminuric” subjects are associated with a commensurate increase in CVD risk^6^.

Unfortunately, the prevalence of smoking in most Middle East and North Africa (MENA) region countries is high, undoubtedly impacting the incidence, prevalence, and rate of progression of CVD in this region.

Since subclinical endothelial injury is the principal mechanism through which smoking increases CVD, podocyturia, as a proposed marker of endothelial injury, would be expected to be particularly elevated in smokers. This definitely warrants further investigation. While the high prevalence of smoking may have increased the prevalence and rate of progression of CVD in our cohort, it does not diminish the predicative power of podocyturia in these subjects. In fact, it may accentuate it.

In our first validation study, we examined the clinical factors associated with finding obstructive coronary artery disease and podocin mRNA elevation among 108 consecutively enrolled patients for a coronary cardiac catheterization. The objective here was not to predict the occurrence of CVD, but rather to test whether the degree of podocyte shedding correlated with the presence and severity of coronary obstruction, thus providing supporting evidence that podocyturia is indeed a marker of endovascular injury. Our findings were similar to the developmental study, in those patients with history of diabetes and a higher mean HBA1C had an increase in podocin mRNA (51% vs 29%, *p* = 0.02; 7.11 ± 1.69 vs 6.2 ± 1.18, *p* = 0.015, respectively). In addition, the significant association between a greater number of diseased vessels and increased podocin mRNA expression (1.89 ± 2.00 vs 1.12 ± 1.47, *p* = 0.03) validates podocyturia as a systemic endothelial/vascular injury marker, as was predicted in the developmental study. Furthermore, in the MESA sample, patients who were diabetic and had higher BMI were more likely to have a greater level of podocyturia, establishing the hypothesis of earlier endothelial dysfunction in patients with metabolic syndrome.

Moreover, our finding that among those who were not taking ACEi/ARB, those with mRNA > 3 were found to have a greater risk for obstructive disease, is novel, yet understandable (OR = 3.31; 95% CI 1.13–9.69). The protective efficacy of ACEis and ARBs on kidney function has been well established in the literature^6,31–33^. Similar results were shown by Ruggenenti et al*.*, who found that patients who were on ACEis had a significantly much lower risk of major adverse cardiovascular events at any level of albuminuria compared to those who were not on ACEis^6^. Moreover, since podocyturia precedes proteinuria, it can be used to measure progression and response to therapy before the development of proteinuria^23,24,34–36^.

The protective effects of the ACEis and ARBs arise from their mechanism of action on lowering blood pressure and the glomerular capillary pressure^37–39^. Interestingly, Durvasula et al.^40^ examined the mechanism by which Angiotensin-ll leads to disruption of mice podocytes exposed to stretching (stress stimuli) versus controls. Compared to controls, stretched podocytes expressed significantly higher expression of Angiotensin-ll levels (*p* < 0.05), a five-fold increase in Angiotensin-ll receptor mRNA expression (*p* < 0.05), and a higher apoptotic rate (2.5 times increase, *p* < 0.001)^40^. In a subgroup analysis, patients with a history of MI, PCI, or CABG, having mRNA levels > 3 were found to have 4.67 times more significant risk of obstructive coronary disease than those with no history. These results echo those of the developmental study that found that after a 7-year follow up, there was a progressive increase in podocin and nephrin mRNA levels (*p* < 0.001). In addition, patients who developed CVD on follow up, had a significant progressive elevation in the podocin and nephrin mRNA levels, highlighting the efficacy of these biomarkers in predicting CVD events as compared to the predictive value of albuminuria [Hazard ratio (HR) for podocin, nephrin and AER was 15.9, 95% CI 6.1–41, 7.61, 95% CI 3.75–15.5, and 1.17, 95% CI 1.01–1.36, respectively].

The subpopulation of patients studied in the MESA cohort provided further evidence, in yet a third population of mixed ethnicities, that podocyturia is indeed a biomarker that tracks remarkably strongly with traditional risk factors for CVD. Studies are currently underway to expand this observation to the entire MESA population and examine the predictive value of podocyturia for the development of CVD.

In conclusion, our studies highlight the potential usefulness of urinary podocyte mRNA quantitation as a surrogate marker for systemic silent microvascular injury. The principal benefit of this assay would be its capacity to identify individuals destined to develop moderate albuminuria and vascular disease several years before either of these outcomes is detectable by present methodologies. Compared to AER, podocyturia may be a more accurate biomarker for the presence of pre-clinical (silent) systemic vascular injury, subsequently manifesting as overt cardiovascular events. In addition, podocyturia may capture at-risk individuals several years earlier than albuminuria, potentially providing a valuable opportunity for preventive interventions. We contend that, pending standardization of urinary podocyte mRNA measurements and independent verification in other cohorts, prospective studies, and other disease states, a clinically applicable assay for podocyte-specific mRNAs may potentially replace AER as the best predictor of systemic vascular injury. Should this be the case, it will be due to the remarkable inherent capacity of glomerular endothelial cells to “report” (through podocyturia) on the health of the systemic endothelium. “Amplification” of the injury “signal” by the large surface area of the glomerular endothelium, the high value of the GFR (180 L/day), and the capacity to detect injury markers in the urine, suggest a novel role for the renal glomerulus as an endogenous “endotheliometer”, providing crucial information on the presence, severity and progression of pre-clinical systemic vascular injury.

### Limitations

Despite the promising and provocative findings, our study has certain limitations that may reflect on its generalizability. Specifically, our study population consisted of a relatively small number of Type II diabetic patients who had normoalbuminuria at enrollment, or who underwent coronary angiography. The applicability of the claimed superiority of podocyturia compared to AER to non-diabetics and the elderly may then be questioned. Our choice of the diabetic population in the developmental study was in fact purposeful, since we expected accelerated endothelial injury compared to non-­diabetics, and hence a relatively more frequent and earlier onset of CVD within 7 years. It is important to note that our study was meant to be a proof of concept proposing novel biomarkers of vascular injury, and hence CVD, prompting a more rigorous assessment of these biomarkers in future studies. The dramatic statistical differences observed even with this small number of patients provides compelling evidence for the underlying hypothesis. We expect that the correlation between podocyturia and systemic vascular injury will be present in non-diabetics as well. This too awaits future more extensive prospective studies.

## Methods

This study was performed in three independent patient cohorts: Cohort A (development data set)—Type II diabetes mellitus patients with longitudinal follow-up over seven years from Hamad General Hospital, Qatar; Cohort B (first validation data set)—suspected coronary artery disease (CAD) patients admitted for coronary angiography at the American University of Beirut Medical Center (AUBMC), Lebanon; and Cohort C (second validation data set)—subgroup of subjects enrolled in the Multi-Ethnic Study of Atherosclerosis (MESA) in the United States.

### Study approvals and ethics declarations

This study was approved by the institutional research boards (IRB) of Hamad General Hospital, Doha—Qatar, AUBMC—Lebanon, and Johns Hopkins University Hospital—United States. Informed consent was secured from study subjects to use their leftover urine samples and to review their medical charts for blood/urine test results and for all relevant clinical diagnoses" (Subject Ethical Approval Research Protocol #11313/11 and IRB approved protocols: IM.AA.07 and IM.KB1.03). All methods were carried out in accordance with relevant guidelines and regulations in accordance with the Declaration of Helsinki.

### Type II diabetes mellitus patients—development cohort A

#### Subjects

A total of 106 study subjects (age: 25–89 years; men/women: 46/60) were selected from the Type II diabetes mellitus patients enrolled for biomarker studies at Hamad General Hospital and followed between 1999 and 2014. Subjects were included: (1) if complete follow up visit data (clinical data and urine samples) were available for at least seven years following their initial visit; (2) if not microalbuminuric at baseline (Visit 1). Microalbuminuria was defined as AER < 2.26 mg/mmol (< 20 µg/mg) albumin/creatinine; and (3) no history of CVD. A diagnosis of hypertension at Visit 1 (n = 17) was not considered CVD in this study.

#### Sample collection and storage

Urine and blood samples were collected during Visit 1 along with a complete medical history and physical examination. Subjects were generally seen at 6 months intervals. Follow-up visits were reviewed for approximately 7 years after Visit 1 to obtain interval history, physical examination, and blood and urine test results at each visit. Clinical data, including new-onset CVD, as well as urine and blood samples were analyzed from Visit 1, Visit 2 (mean follow up after Visit 1 of 3.8 years; SD 0.6 years) and Visit 3 (mean follow up after Visit 2 of 3.2 + / − 0.7 years).

Urine samples were kept at 4 °C (for a maximum of 24 h) until moved to the laboratory where they were kept at − 80 °C for prolonged storage period. The influence of storage temperature and freeze–thaw cycles on urinary RNA stability and integrity was examined at temperatures ranging from room temperature to − 80 °C over 20 days; − 70 °C proved the optimal temperature in terms of future RNA sample stability, since the RNA samples were degraded by almost 50–60% when kept between 4 °C and − 10 °C. RNA degradation was also detected in samples stored at − 20 °C and − 40 °C; no such effects were detected with storage at − 80 °C. Urine samples were analyzed for albumin excretion rates (AER; mg/mmol creatinine) and for podocin and nephrin mRNA (millions of copies/sample).

#### Procedures

##### Volume of urine for assay

We assumed that the samples contained low mRNA levels. Podocin and nephrin mRNA were not detectable in 25–30% of samples starting with < 1 ml of urine. Therefore, 1.7 ml were used in all assays.

##### Urine processing

Urine was centrifuged at 4 °C for 15 min at 4000 rpm (3200 g) on a table-top centrifuge. The supernatant was removed and stored at − 20 °C for protein, creatinine, and other measurements. The urine pellet was suspended in 1.5 ml of cold diethylpyrocarbonate-treated PBS (pH, 7.4) at 4 °C. The pellet material in 1.5 ml PBS was then centrifuged at 12,000 rpm in a mini-centrifuge for 5 min at 4 °C. The supernatant was discarded. 350 μl of RLT buffer was added to the washed pellet, containing β- mercaptoethanol at 10 μl/ml of RLT buffer, according to the manufacturer protocol (RNeasy kit; Qiagen—Germantown, MD). The pellet was suspended in RLT/β − mercaptoethanol buffer and then frozen at − 80 °C for assay.

##### RNA preparation and quantitative RT-PCR assay

The total urine pellet RNA was isolated using the manufacturer protocol (RNeasy Mini Kit; Qiagen—Germantown, MD). Quantitation of the absolute nephrin and podocin, mRNA abundance was performed using the CFX96 Touch™ Real-Time PCR Detection System (Bio-rad, USA) using TaqMan Fast Universal PCR Master Mix, with sample cDNA in a final volume of 25 μl per reaction. TaqMan Probes (Applied Biosystems) used were as follows: human NPHS1 (nephrin gene accession number: Q1KMS5) and NPHS2 (podocin gene accession number: Q9NP85). All data were from 2-μl sample measured in duplicate. Standard curves were constructed for each assay using serially diluted cDNA standards. Assays were accepted only if the r2 > 0.97 for standard curves. Human nephrin and podocin cDNA of known sequence and concentration were used as standards for each assay so that the data could be calculated on number of copy basis for each probe. RNA urine analysis quality, recovery, and stability were performed.

Samples were coded and analyzed with the laboratory operator blinded to the visit number, the subject it belonged to, or whether this subject eventually developed CVD or not.

### Coronary angiography for suspected CAD patients: validation cohort B

#### Subjects

Urine samples were collected and analyzed from 108 consecutive consenting adult patients (mean (± SD) age 63.5 ± 9.71 years; men/women: 83/25) with suspected CAD enrolled from the cardiac catheterization laboratory at AUBMC in 2016 and 2017. Patients were excluded in case of incomplete complete clinical data or if urine samples were not available. The CV endpoint was absence or presence of obstructive CAD defined as 50% or more stenosis of one or more epicardial or branch vessels.

#### Sample collection and storage

In this validation study, we used podocin mRNA [higher prediction rate, as demonstrated from the development cohort studies] to quantify urinary podocyte shedding. Urine samples were assigned a study number that linked them to the clinical information, and were analyzed with the laboratory operator blinded to the subject they belonged to. Levels of podocyturia were correlated with patient demographic and clinical data along with results of coronary angiographic results.

### Patient samples from the multi-ethnic study of atherosclerosis (MESA) study – validation cohort C

#### Study design and patients

The second validation cohort consisted of a sample of 480 subjects enrolled into the MESA study, which included asymptomatic men and women aged 45–84 who were free of cardiovascular disease at recruitment. Participants in the MESA dataset were followed up longitudinally at clinics at Columbia University, New York; Johns Hopkins University, Baltimore; Northwestern University, Chicago; UCLA, Los Angeles; University of Minnesota, Twin Cities; Wake Forest University, Winston Salem^41^. For this study, podocyte mRNA measurements were performed on urine samples from Visit 1 stored at − 80 °C.

### Statistical methods

#### Development cohort A

The probability of CVD was analyzed using Kaplan–Meier survival analysis to compare AER, urinary nephrin mRNA and urinary podocin mRNA using log-rank test. A repeated measure analysis was performed to compare the mean of nephrin mRNA, podocin mRNA and estimated glomerular filtration rate (eGFR; CKD-epi formula) measured at visits 1, 2 and 3 for the sub-cohort of diabetic patients that did (CVD) versus did not (No-CVD) develop a CVD event.

#### Validation cohort B

The primary outcome of interest was finding an obstructive vessel. Continuous variables including levels of podocyturia mRNA (podocin) were expressed as mean ± standard deviation or median (inter-quartile range), as applicable, and compared using the independent t-test. Categorical variables were expressed as counts (percentages) and compared by the chi-square test. Multivariate regression was used to risk adjust for other covariates associated with measured indices of podocyturia and abnormal vessel findings.

#### Validation cohort C

The primary objective was estimating associations between podocyte mRNA copies and clinical risk factors for CVD. Sociodemographic and risk factors for CVD were compared across terciles of podocyturia mRNA copies using ANOVA for continuous variables and chi-square tests for categorical variables. Levels of podocin and nephrin were compared among diabetic and non-diabetic participants using independent t-tests.

In all studies, a *p* value less than 0.05 was used to indicate statistical significance. The Statistical Package for the Social Sciences (SPSS) version 23.0 and SAS software were used for data cleaning, management and analysis.

## Supplementary Information

Supplementary Tables.

## Data Availability

All data generated or analyzed during this study are included in this published article.
