# Supplementary material for: Podocyturia: an earlier biomarker of cardiovascular outcomes
Source: Sci Rep. 2022 Dec 13;12:21563. doi: 10.1038/s41598-022-26162-6 (PMC9747803; doi:10.1038/s41598-022-26162-6)
Supplement: Supplementary file 1 — Supplementary Tables. [file 41598_2022_26162_MOESM1_ESM.docx]

**Table 1 Supplement: Multivariable logistic regression analysis for factors predicting having obstructive coronary artery disease**

| **Vessels (Reference: normal)** | | |
| --- | --- | --- |
| **Variables** | OR (95 % CI) | P-value |
| **RNA** | 1.78 (0.74 - 4.29) | 0.20 |
| **Gender** | 3.59 (1.21 - 10.71) | **0.02** |
| **Age** | 1.51 (0.93 - 2.45) | 0.10 |
| **Diabetes** | 2.88 (1.14 - 7.33) | **0.03** |
| **MI/PCI/CABG** | 2.31 (0.90 - 5.96) | 0.08 |
| Variables included in the model were:  Imposed: RNA (Reference: <3)  Stepwise: Age (increase by 1 unit); gender (reference: female); Drug Ace Inhibitor /ARB (reference: no); Diabetes (reference: no); Hypertension (reference: no); MI /PCI /CABG (reference: no); Cath (reference: no). | | |

**Table 2 Supplement: The association between RNA and Vessels among different groups of patients**

|  | RNA (>3) in association with vessels (obstructive coronary artery disease) | | |
| --- | --- | --- | --- |
|  |  | OR ( 95 % CI ) | P-value |
| **Age** | <63 | 2.47 (0.83 - 7.39) | 0.10 |
|  | ≥63 | 1.77 (0.56 - 5.57) | 0.33 |
| **Gender** | Male | 2.34 (0.93 - 5.84) | 0.07 |
|  | Female | 1.61 (0.31 - 8.32) | 0.57 |
| **Ace Inhibitor /ARB** | No | 3.31 (1.13 - 9.69) | **0.03** |
|  | Yes | 1.45 (0.44 - 4.71) | 0.54 |
| **Diabetes Status** | No | 1.40 (0.52 - 3.78) | 0.51 |
|  | Yes | 2.78 (0.65 - 11.90) | 0.17 |
| **MI /PCI /CABG** | No | 1.63 (0.62 - 4.28) | 0.32 |
|  | Yes | 4.67 (0.99 - 22.01) | 0.05 |
